# Volatile Organic Compounds as Early Detection Indicators of Wheat Infected by *Sitophilus oryzae*

**DOI:** 10.3390/foods13213390

**Published:** 2024-10-24

**Authors:** Xinjie Liu, Haixin Jiang, Haoqi Xu, Sijia Shang, Dianxuan Wang, Yueliang Bai, Fangfang Zeng

**Affiliations:** 1 School of Food Science and Technology, Henan University of Technology, Zhengzhou 450001, China; 17513167865@163.com; 2 National Grain Industry (Storage Insect Pest Control) Technology Innovation Center, School of Food and Strategic Reserves, Henan University of Technology, Zhengzhou 450001, China; 3 Grain Storage and Logistics National Engineering Technology Research Center, School of Food and Strategic Reserves, Henan University of Technology, Zhengzhou 450001, China

**Keywords:** stored pest, rice weevil, infested wheat, volatiles, early detection

## Abstract

The rice weevil, *Sitophilus oryzae* (L.), is a major pest that poses a considerable threat to grain safety storage. Early detection is of great significance in reducing grain losses. Studies have demonstrated that pest infestation causes alterations in grain volatiles, potentially indicating the presence of pests. In this study, we detected volatile organic compounds (VOCs) in non-infected and pest-infected wheat on the 3rd, 9th, 17th, 22nd, and 40th days, corresponding to the developmental stages of the rice weevil at the egg, young larval, old larval, pupal, and adult stages, respectively. A total of 126 VOCs were identified, including 96 hydrocarbons, 7 alcohols, 5 aldehydes, 9 ketones, 9 esters, and 18 other compounds, 62 of which are newly produced compared to non-infected wheat. Six characteristic volatiles, namely dodecane, pentadecane, hexadecane, heptadecane, 2, 6, 10-trimethylpentadecane, and squalene, may be related to the degradation of lipids and the expression of wheat stress tolerance and underwent significant changes as infestation progressed, according to the VIP value. This study assists in interpreting the effects of rice weevil infestation on wheat at the metabolic level and establishes a foundation for storage inspection based on VOC analysis.

## 1. Introduction

Stored pests are a critical issue for the global food supply, as their grain feeding leads to weight loss and nutrient loss, and their metabolites can lower grain quality and lead to microbial proliferation that causes mold and mildew [1]. Globally, grain losses during storage contribute 15–25% of total losses, and the proportion of post-harvest losses produced by pests ranges from 9% in developed countries to 20% or more in developing countries [2]. Among stored grain pests, internally infesting insects are considered the most destructive, primarily due to the extensive damage they cause by consuming large portions of the grain kernel during their development [3]. The rice weevil, *Sitophilus oryzae* (Linnaeus), is a crucial stored grain pest. Females lay their eggs within seeds during storage or the pre-harvest period, leading to grain that is already infested when harvested. These eggs then hatch during storage and the immature stages develop inside the kernels, causing substantial economic losses [4,5]. Due to the weevil’s hidden behavior, infestations are difficult to detect and are not easily eliminated during processing. Currently, early detection of hidden pests in grain is challenging, and existing detection methods mainly include X-ray, acoustic, whole grain flotation, ninhydrin approaches, etc. However, these methods are either cumbersome, labor intensive, susceptible to environmental influences, inefficient in detecting immature stages of insects and internal infestation, or not sensitive enough [6,7]. Novel methods of sample collection, insect detection, and data analysis should be developed to report the real-time quality of stored grains, allowing fast and appropriate management decisions.

Pest detection technology based on volatile compounds has many advantages, such as high sensitivity, non-destructiveness, real-time monitoring, not being limited to specific conditions, etc. [8]. A potential early pest infection detection approach is to investigate the air samples from a grain mass to detect specific volatile compounds (VOCs) released by insect-infested grains, which can be biomarkers for monitoring grain pest infestation. Studies have demonstrated that during grain storage, the physiological behaviors of grain storage pests, including feeding and excretion, the growth and reproduction of microorganisms, and the volatiles of insects and microorganisms cause changes in the volatiles present in the ambient gas within the grain silo [8]. Volatiles can be utilized as an indicator of the quality of stored grain [9]. Senthilkumar et al. [10] demonstrated that the number of volatiles produced by adult *Tribolium castaneum* increases with increased insect density. Similar results were found in *S*. *oryzae* infestation in stored rice grain, as significant changes in uric acid and protein content were found in rice grains infested with rice weevils via an electronic nose sensor [11]. Detecting the dynamic changes of volatiles in grain silos can help us understand the storage environment and determine the contamination of grain in silos by pests [12,13]. Zhang et al. [14] demonstrated that over more than 30 days of infestation by *S. oryzae*, a high variability of volatile fractions was detected in wheat storage environments. However, whether VOCs change in the early infestation and whether they can be used as detection indicators are still unknown.

Commonly used volatile collection methods include headspace gas flow collection and solid-phase microextraction (SPME). Headspace gas flow can overcome damaging of the sample during collection and can simulate the collection of wheat volatiles in the grain silo under natural conditions [15,16]. Headspace gas flow collection and gas chromatography-mass spectrometry (GC-MS) have strong enrichment ability for samples, are easy to operate, and have high sensitivity [17]. Thus, in this study, headspace gas flow collection and GC-MS were used to analyze the volatile compounds in healthy wheat, wheat infested with rice weevils at different developmental stages (egg, young larval, old larval, pupal, and adult stages (when the wheat has been hollowed out by rice weevils)), to investigate the effects of early rice weevil larvae infestation on volatile compounds in wheat and to compare differences in volatile compounds between healthy and infested wheat through Partial Least Squares Discriminant Analysis (PLS-DA) analysis. This study aims to explore the feasibility of using volatile detection to characterize infestation by rice weevils to maintain the quality and safety of grain.

## 2. Material and Methods

### 2.1. Insect Rearing

Rice weevil test samples were cultured for over ten generations in the Insect Laboratory of Henan University of Technology under an ambient temperature of 28 ± 1 °C and relative humidity of 70 ± 5%. Insect individuals were reared on whole wheat kernels.

### 2.2. Sample Preparation

Wheat was washed and dried until its moisture content reached 12.5%. Six distinct damage phases were set as our experimental conditions (the 3rd, 9th, 17th, 22nd, and 40th days after oviposition, corresponding to the egg, larva, pupae, and adult stages of the rice weevil, respectively [18]). Six biological replicates were used for each treatment. Specifically, for each replicate, 1000 adult rice weevils were placed in glass bottles containing 400 g of wheat at an ambient temperature of 28 ± 1 °C and relative humidity of 70 ± 5%. The adults were then removed the next day to obtain wheat with eggs for subsequent cultivation. After 3, 9, 17, 22, and 40 days of cultivation, 100 g of wheat were taken out for volatile analysis, respectively. Uninfected wheat was kept under the same conditions as the control group.

### 2.3. Volatile Collection

Wheat volatiles were obtained using the dynamic headspace adsorption method. Specifically, an air pump (ACO-002 electromagnetic air pump, Sensen, Zhengzhou, China) was used to evacuate the dynamic headspace adsorption instrument (Hongyi Instrument Company, Wuhan, China) (homemade glass jar with a total volume of 300 mL, with two connections added to the lid: one was designed to connect with filtered clean air, while the other was either linked to the absorption column or left open to the ambient air) compartment for 2 h to remove the odor. The adsorption column was cleaned with hexane(Sigma-Aldrich, St. Louis, MO, USA) at least three times and installed in the dynamic headspace adsorption instrument to blow for 1 h; the infested grains containing different developmental stages of rice weevil were placed into each instrument compartment separately for each stage, and the air pump was turned on for 24 h. Hexane was used as an eluent to elute the adsorption column more than three times, and then nitrogen blowing was used to remove volatiles from the column. The *n*-hexane eluent dissolved in the volatiles was concentrated to 0.20 mL via nitrogen blowing, stored in a glass sample bottle, and maintained at −80 °C for analysis by GC-MS.

### 2.4. GC-MS Analysis

A GC-MS-QP 2010Ultra (Shimadzu, Kyoto, Japan) was employed for chemical analysis. An HP-5MS capillary column (30 m × 0.25 mm, 0.25 μm) was utilized. The heating program was as follows: the initial temperature was held at 40 °C for 4 min, increased to 200 °C at 5 °C /minute, and maintained for 2 min; then it was increased to 280 °C at 20 °C /minute for 4 min. The temperature of the inlet port was 250 °C and the carrier gas was He at a flow rate of 1.0 mL/minute.

Mass spectrometer conditions were as follows: the inlet temperature was 250 °C, the interface temperature was 280 °C, the ion source was EI source, the temperature was 230 °C, and the electron energy was 70 eV. The full scanning mode was used for information acquisition, and the mass spectrometry mass scanning range was *m/z* 50 to 550.

### 2.5. Data Processing

All samples were evaluated three times, and each unknown volatile compound in the sample was characterized by computer search and search of the mass spectrometry library for matching. The volatile components with a statistical match of over 80% were used as identification results [14]. Quantitative analysis of compounds aligned with the peak area normalization method of retrieval, analysis, and calculation to find the ratio of peak area to total peak area for the volatile substances identified. The relative levels of volatiles were determined by the ratio of the peak area of detected volatiles to the total peak area, and the results were presented as relative content ± standard error. Correlation data were visualized using a heat map made with OriginPro 2021 software. Principal component analysis (PCA) and partial least squares-discriminant analysis (PLS-DA) were performed using MetaboAnalyst 5.0 software.

## 3. Results

### 3.1. Analysis of Volatile Components in Rice Weevil-Infested Wheat over Different Stages

**Figure 1:**
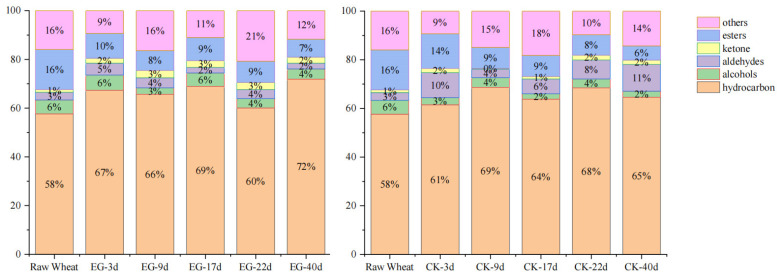
Histogram of volatile percentage stacking at each stage for experimental and control groups. CK-3d, 9d, 17d, 22d, and 40d refer to wheat samples stored naturally for 3, 9, 17, 22, and 40 days, while EG-3d, 9d, 17d, 22d, and 40d refer to wheat samples after 3, 9, 17, 22, and 40 days of infestation by rice weevil.

**Figure 2:**
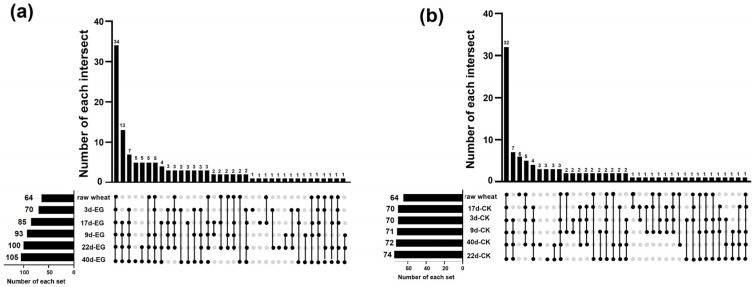
Upset plots of volatiles at each stage. (**a**) experimental group: EG-3d, 9d, 17d, 22d, and 40d refer to wheat samples after 3, 9, 17, 22, and 40 days of infestation by rice weevil; (**b**) control group: CK-3d, 9d, 17d, 22d, and 40d refer to wheat samples stored naturally for 3, 9, 17, 22, and 40 days. “The number of each set” denotes the total number of volatile species detected for each sample. “The number of each intersect” on the y-axis represents the number of shared volatiles detected by multiple databases; a point on the horizontal axis represents the number of unique volatiles detected by the database; multiple point lines on the horizontal axis represent the number of shared volatiles identified by multiple databases along the line.

As presented in Supporting Information Table S1, analysis of volatile components in wheat infested with rice weevil over different stages and control samples demonstrated that 126 compounds were identified, encompassing 96 hydrocarbons, 7 alcohols, 5 aldehydes, 9 ketones, 9 esters, and 18 other compounds, among which 64 were found in untreated wheat and 62 increased following infestation with rice weevil. According to the percentage stacking histogram (Figure 1), hydrocarbon compounds accounted for the highest proportion in untreated wheat and rice weevil-infested samples across all stages, with relative levels of 58% to 72%. With increased infestation time, their relative contents fluctuated and increased. The relative levels increased as much as 14% from untreated wheat to wheat infested with rice weevil for 40 days. The levels of contents of esters decreased more significantly. The relative content of esters decreased from 16% to 6%, while the relative content of alcohols decreased only slightly. The relative levels of aldehydes and ketones were lower, and there was no obvious change. According to our analysis (Figure 2), the volatile species in both EG-3d and CK-3d samples included six more types than the original wheat. EG-9d volatile species included 23 new types compared to EG-3d, while the number of CK-9d volatile species was only one type higher than CK-3d. The number of volatile species in EG-17d decreased by eight types over eight days, while in CK-17d, it decreased by one type. The total number of EG-22d volatile species was 15 higher than EG-17d, while CK-22d added four new types compared to CK-17d. The total number observed in EG-40d included five new types compared to EG-22d, while CK-40d decreased by two types compared to CK-22d. Overall, the number of volatile species in the experimental group changed more than that of the control group, consistent with the pattern of change observed (Figure 2).

### 3.2. Principal Component Analysis (PCA)

**Figure 3:**
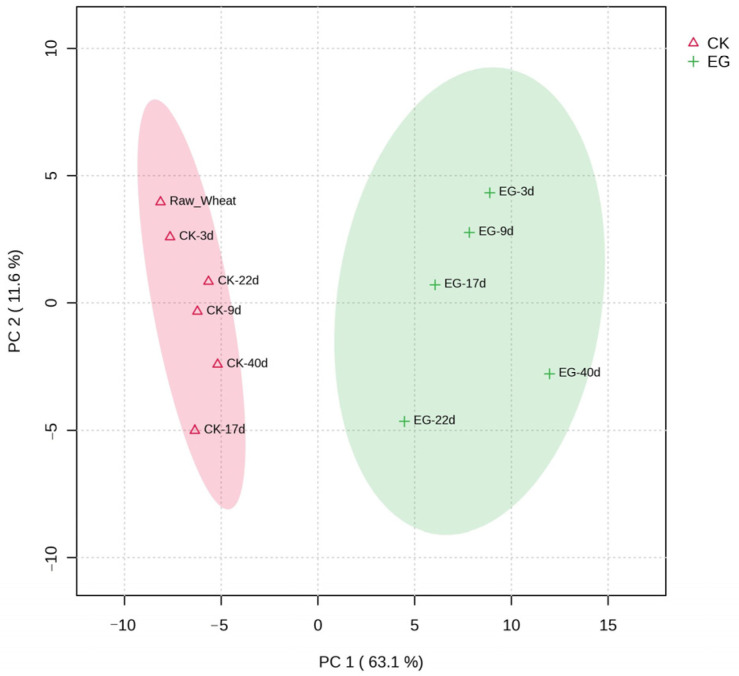
Scores of volatiles at each stage of volatile analysis between wheat and rice weevil infested wheat. CK: control group, CK-3d, 9d, 17d, 22d, and 40d refer to wheat samples stored naturally for 3, 9, 17, 22, and 40 days; EG: experimental group, EG-3d, 9d, 17d, 22d, and 40d denote wheat samples after 3, 9, 17, 22, and 40 days of infestation by rice weevil.

PCA is a common information statistical method to downscale the raw sample data obtained through a multidimensional matrix sequence [19]. To investigate the GC-MS results more deeply, we conducted PCA on the data of the relative contents of the volatiles from different samples (Figure 3). The first principal component contributed 63.1%, the second 11.6%, and the third 7.8%, which cumulatively accounted for 82.5% of the original variables, and the 95% confidence ellipse of the experimental group samples and the control samples did not intersect. The rice weevil-infected wheat samples and control samples were clearly separated in PCA score plots. The rice weevil-infected grain samples were almost entirely located in the region with positive scores, whereas the cluster of control samples was well defined by negative PC1 scores, suggesting that the relative content of volatiles changed between the control samples and the rice weevil-infected wheat samples. The data points of the control group were distant from the experimental group. The experimental group was relatively more dispersed, reflecting the significant difference in the volatiles of uninfested and infested wheat samples in the same time period. Additionally, this indicated a more significant change in volatile fractions in infested wheat over different times.

### 3.3. Partial Least Squares Discriminant Analysis (PLS-DA)

**Figure 4:**
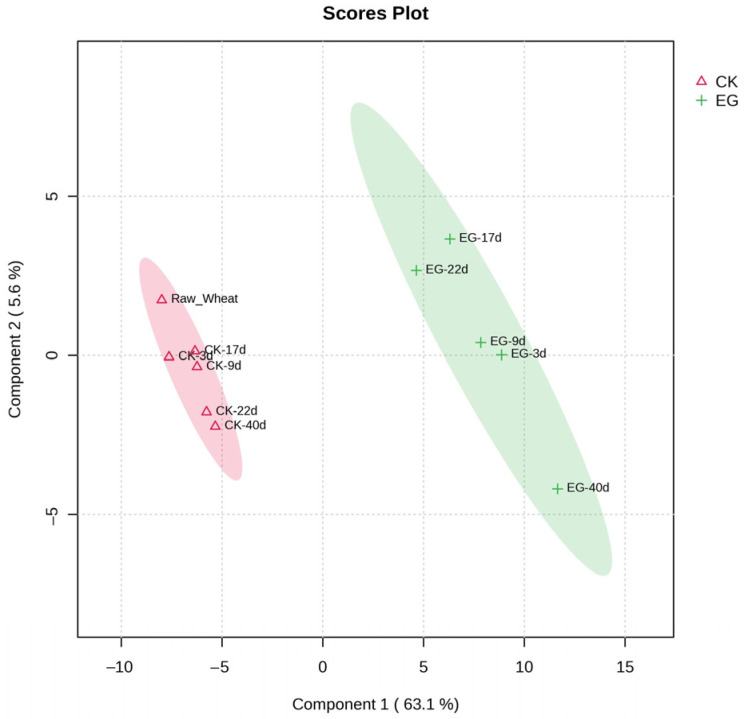
Plot of partial least squares discriminant analysis scores of volatiles at each stage between wheat and rice weevil-infested wheat. CK: control group, CK-3d, 9d, 17d, 22d, and 40d refer to wheat samples stored naturally for 3, 9, 17, 22, and 40 days; EG: experimental group, EG-3d, 9d, 17d, 22d, and 40d refer to wheat samples after 3, 9, 17, 22, and 40 days of infestation by rice weevil.

PLS-DA is a multivariate, supervised statistical analysis method that filters out irrelevant variables to maximize differences between groups [20]. To better reflect the differences in volatile fractions of wheat following rice weevil infestation, the data were processed by PLS-DA scores of volatiles (Figure 4). The fitted model had *R*^2^(X) = 0.944, *R*^2^(Y) = 0.992, and *Q*^2^ = 0.904, and the three fitted parameters were all higher than the recommended value of 0.5 and were close to 1, indicating that the model had good interpret-ability and predictability without overfitting. The data points of each wheat volatile at varying infestation times were clustered in two regions. The 40-day infested hollowed wheat volatiles data points were distant from the control data points and were located at the bottom edge of the 95% confidence ellipse of the experimental group, indicating that these volatile components differed from the rest of the samples in the experimental group, consistent with the previous inference.

**Figure 5:**
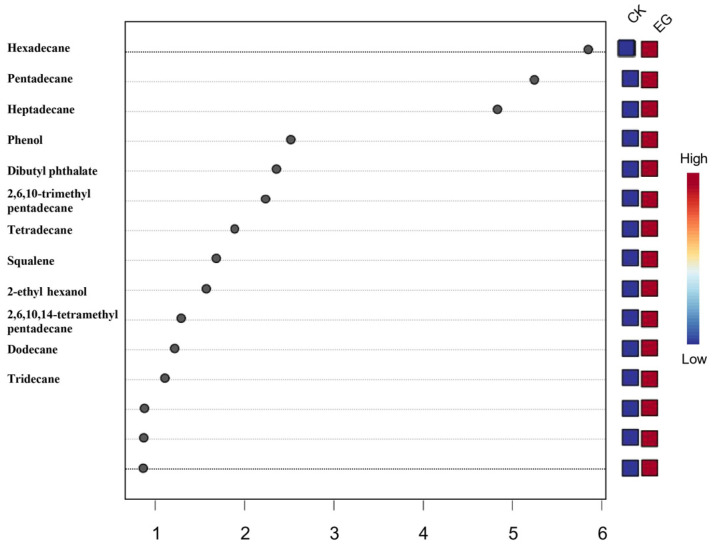
Projected importance (VIP) results of volatile variables for each wheat sample. The horizontal coordinate is VIP (Variable Importance Projection) and reflects the magnitude of the variable’s contribution to the model’s overall fit and classification ability.

A total of 12 substances with variable importance projection (VIP) values over 1 (recommended threshold) in the PLS-DA model data were identified (Figure 5): hexadecane (*p* = 4.18 × 10^−6^), pentadecane (*p* = 8.24 × 10^−6^), heptadecane (*p* = 2.66 × 10^−5^), phenol (*p* = 1.45 × 10^−4^), dibutyl phthalate (*p* = 0.0513), 2, 6, 10-trimethylpentadecane (*p* = 2.01 × 10^−6^), tetradecane (*p* = 0.0897), squalene (*p* = 0.0409), 2-ethylhexanol (*p* = 7.72 × 10^−6^), 2, 6, 10, 14-tetramethylpentadecane (*p* = 0.0965), dodecane (*p* = 0.0467), and tridecane (*p* = 0.24) (the *P*-value is the parameter used to determine the results of the hypothesis test, *p* > 0.05 indicates no significant difference). Phenol, dibutyl phthalate, and 2-ethylhexanol levels depended on the storage environment or experimental conditions. The differences in dibutyl phthalate, tetradecane, 2, 6, 10, 14-tetramethylpentadecane, and tridecane were not obvious before and after infestation with the rice weevil (*p* > 0.05), while the other six substances (dodecane, pentadecane, hexadecane, heptadecane, 2, 6, 10-trimethylpentadecane, and squalene) could be used as characteristic volatiles of rice weevil-infested wheat. The relative levels of pentadecane and hexadecane changed most significantly at the earliest stage of wheat infestation, increasing from 2% to 11% during the first three days of infestation. Their contents can be employed as a criterion for determining whether or not wheat is infested by rice weevil at the earliest stage.

### 3.4. Hierarchical Clustering Analysis of Characteristic Volatile Substances

**Figure 6:**
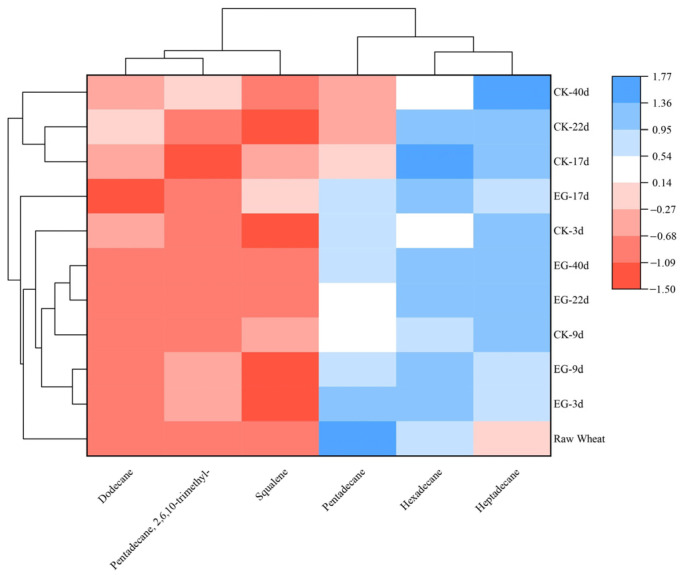
Hierarchical cluster analysis heat map of volatiles characterizing rice weevil-infested wheat. The horizontal axis denotes the volatile, and the vertical axis is the name of the sample. Colors indicate high or low expression: red (negative) indicates low expression and blue (positive) indicates high expression. The clustering trees on the top and left depict the clustering of volatiles and samples.

The clustering heat map reflects the dynamic alterations of substances more intuitively. In this study, six characteristic volatile compounds were identified and clustered in a hierarchical manner by a complete chaining method (Figure 6). After 17, 22, and 40 days, uninfected wheat was clearly separated from other samples, indicating that the volatile components of naturally stored wheat might change around 17 days. In contrast, the infested wheat and the original wheat were clearly separated, and the volatiles of the two groups of wheat infested with the rice weevil for 3 and 9 days were clustered. The samples infested for 22 and 40 days were clustered, indicating that the volatile components of the rice weevil are similar, suggesting a dynamic change of the substances. According to the color of the color block, the relative contents of the five characteristic volatiles in the experimental group generally increased, and the control group samples were different from the original wheat volatiles, but the difference was small. The expression of each substance also varied, with the first three volatiles on the horizontal axis having low expression and the other three having high expression, so they were categorized into different groups, suggesting that these compounds may have proximate biological functions or participate in metabolic activities in the wheat storage environment [21].

## 4. Discussion

Compared with the 64 volatiles detected in rice weevil-infested wheat samples using solid-phase microextraction by Zhang et al. [14], a total of 126 volatiles were identified by headspace gas flow collection coupled with GC-MS in the present study, with 48 more hydrocarbons and the highest content. This indicated that headspace gas flow collection is highly sensitive. Comparing wheat volatiles when infested with rice weevil with the volatile components of each sample from natural storage, the differences between the samples infested with rice weevil were mainly reflected in the content of n-alkanes. The differences in the remaining categories of compounds were reduced, mirroring a study by Shan et al. [20].

Hydrocarbon compounds are the predominant volatile components in wheat initially. Following infestation by the rice weevil, the number of detected hydrocarbon compounds rose by 35 additional varieties (Table S1). Among them, compounds such as *n*-tridecane, *n*-hexadecane, and other alkanes increased slowly in relative content at 3, 9, 17, and 22 days of infestation by rice weevils, and all of them were different from the control group (*p* < 0.05) and were significantly different at 40 days (*p* < 0.01). Among the six characteristic volatile compounds, dodecane, pentadecane, hexadecane, and heptadecane, as *n*-alkanes, are components of waxes in the surface layer of plants. The reason for their slight increase in the pre-infestation period may be due to the degradation of lipids in wheat [22], as well as the increased secretion of waxes due to increased resistance [23]. Under experimental conditions, rice weevils entered the adult stage around 30 days, and the alkanes and olefins in the epidermal lipids of adult weevils may have caused the substantial increase in the relative content of the hydrocarbon compounds [24]. The significant increase in the content of 2, 6, 10-trimethylpentadecane at 40d may be related to this. In wheat samples infested for 22 days, the relative levels of hydrocarbons decreased significantly, likely due to rice weevils entering the pupal stage at about 22 days, and their life activities were weakened, reducing damage to wheat. In addition to these reductions, the types and contents of the remaining compounds are still subject to the influence of the metabolism of the wheat itself and continue to increase [25].

Squalene is widely distributed in nature, improving the body’s defense function and repairing damage [26], and it has cardiovascular protection and antitumor and antioxidant activities [27,28]. In all samples, the olefin species were reduced relative to alkanes, and the relative content of squalene first increased sharply and then decreased. The increase in its relative content may be due to the feeding of rice weevils producing increased wheat resilience. The production of squalene repairs the damage caused by the late infestation and may cause a loss of nutrition due to the seriousness of wheat breakage.

Most of the differences in the relative levels of aldehydes, ketones, and some esters are due to the hydrolysis and oxidation of lipids. Although the lipid content of wheat is lower than proteins and starch, lipids are more susceptible to degradation during storage, and aldehydes and ketones are easily volatilizable, leading to unpleasant odors and reduced wheat quality [29,30,31]. After rice weevil infestation, the number of aldehyde compound species increased by two, and ketone compound species increased by eight. However, the relative levels of the two types of compounds changed less, demonstrating a trend of increasing first and decreasing later. Literature indicates that straight-chain aldehydes such as nonanal are generated by the oxidative decomposition of oleic and linoleic acids [32], while dodecanal and others are degraded from fatty acids by hemolytic cleavage of chemical bonds. Ketone compounds with the highest percentage of 6-methyl-5-hepten-2-one content experienced the most significant changes, are linked to the production of pyruvic acid, and are also a characteristic volatile in yellowing rice. After infestation, there was an increase of four esters in wheat, and the differences in the relative content of esters may be due to the ongoing metabolism of amino acids and the fatty acid lipoxygenase process, and a small number of esters are also produced by aldehyde esterification [33].

Alcohols in cereals are one of the products of oxidative decomposition of unsaturated fatty acids and are formed by further decomposition of aldehydes [33]. The number of alcohols identified in wheat infested with rice weevil increased by four, and although their relative levels tended to decrease, except for the phytol content, the contents of other substances changed more irregularly and were not detected across several samples. The trace increase in phytol may be due to the hydrolysis of chlorophyll in wheat.

Phenol and dibutyl phthalate are considered to be caused by atmospheric pollution, pesticide contamination, exposure to plastics, and exposure to polymer materials in the experimental apparatus during the growth, packaging, and transportation of wheat. Phenol is a common aromatic pollutant in paper, printing, plastics and other industrial processes [34,35,36]. The highest relative content of phenol detected in our study was 3.95%. Dibutyl phthalate, as well as four phthalate esters detected in all samples, is commonly used in polyvinyl chloride materials and is a common environmental hormone in the living environment [37,38,39].

The difference in volatiles of wheat stored under natural conditions is primarily caused by lipid degradation, while the infestation by the rice weevil aggravated lipid degradation to a certain extent. The metabolites produced by weevils triggering enhanced wheat resistance may be the reason for the difference in volatile components.

VOC monitoring technologies for the early detection of grain pests, particularly with custom-made VOC sensors, have gained significant attention. While there have been advancements in real-time monitoring of grain quality in the bins, there are still challenges to overcome in VOC monitoring technologies for early detection of grain pests, such as identifying volatile markers of various pests invading grains, advancing VOC monitoring to improve detection performance, and developing multifunctional integration of sensors [40]. Here, we studied the changes in volatile compounds of wheat infected with rice weevils; however, under actual storage conditions, there are also many factors that affect the production of volatile compounds, such as environmental temperature, humidity, microorganisms, other pest species, grain varieties, etc. [8]. Therefore, there is still a lot of work to be done in the application of volatile compound detection for monitoring. Despite the challenges that need to be addressed, researchers are actively working towards developing VOC sensors and devices with excellent performance for real-time monitoring in grain silos. With further advancements in sensor technology and interdisciplinary collaboration, we can expect significant progress in this area to improve pest management and sustainable practices.

## 5. Conclusions

This research investigated the differences in the components of volatile substances produced at different times after infestation by rice weevils. Among the 126 volatile compounds identified by GC-MS, hydrocarbons had the most types and the highest content percentage. With the increased duration and prolongation of infestation time, hydrocarbons continued to accumulate, and the total content exhibited an increasing trend. The relative content of esters and alcohols showed a decreasing trend, and the changes in alcohols and aldehydes were not obvious. It can be preliminarily inferred that the difference in volatiles of wheat stored under natural conditions is mainly caused by lipid degradation, while the infestation by the rice weevil exacerbated lipid degradation to a certain extent, and the metabolites generated by its triggering of enhanced wheat resistance may be the main reason for the difference in volatile components. Additionally, the results of PCA, PLS-DA analysis, and the cluster heat map clearly demonstrated significant differences in wheat volatiles before and after rice weevil infestation over time. Through further analysis, the relative levels of dodecane, pentadecane, hexadecane, heptadecane, 2, 6, 10-trimethylpentadecane, and squalene, including their large changes before and after rice weevil infestation, indicate that they can be used as biochemical markers. Increases in dodecane, pentadecane, hexadecane, heptadecane, and squalene can be used as indicators of wheat being infested by rice weevil larvae, and the sudden increase of dodecane, pentadecane, hexadecane, and heptadecane and decrease in squalene can be indicators that rice weevils have entered into adulthood. This study helps to deepen the understanding of wheat volatiles before and after infestation by pests and verifies that headspace gas flow collection and GC-MS can be efficiently used to extract volatile compounds from wheat. Early detection of grain pests based on VOC monitoring technologies is an emerging and promising field that holds great potential for the advancement of precision control. This can be applied to the quality testing of wheat and determining whether the kernels have been attacked by pests during storage and has diverse prospects for cross-border grain trade inspection. Our future work will focus on following aspects: establishing volatile compound databases for different grains under different storage conditions both in laboratory simulation and actual warehouse conditions to identify volatile markers and then developing customized VOC sensing arrays and detection models integrated with intelligent grain detection systems for precision agriculture to achieve on-site, rapid, and real-time monitoring of grain pest infestations.

## Data Availability

The original contributions presented in the study are included in the article/Supplementary Materials, and further inquiries can be directed to the corresponding author.

## Supplementary Materials

The following supporting information can be downloaded at: https://www.mdpi.com/article/10.3390/foods13213390/s1, Table S1. Volatile components of wheat and rice weevil-infested wheat at various stages of the process.
