# Supplementary material for: Volatile Organic Compounds as Early Detection Indicators of Wheat Infected by Sitophilus oryzae
Source: Foods. 2024 Oct 24;13(21):3390. doi: 10.3390/foods13213390 (PMC11545270; doi:10.3390/foods13213390)
Supplement: Supplementary file 1 [file foods-13-03390-s001.zip › foods-3261157-supplementary.pdf]

**Table S1.** Volatile components of wheat and rice weevil-infested wheat at various stages of the process †.

| **Compound Class** | **Compound Name** | **Relative content of compounds/%** | | | | | | | | | | |
|---|---|---|---|---|---|---|---|---|---|---|---|---|
| | | **raw wheat** | **3d-EG** | **3d-CK** | **9d-EG** | **9d-CK** | **17d-EG** | **17d-CK** | **22d-EG** | **22d-CK** | **40d-EG** | **40d-CK** |
| | 10-Methyleicosane | - | 0.90±0.01 | - | - | - | 0.29±0.07 | - | - | - | 0.87±0.13 | - |
| | 11-(1-Ethylpropyl) heneicosane | - | - | 0.28±0.04 | - | 0.59±0.02 | - | 0.19±0.01 | 0.29±0.01 | 0.29±0.04 | 0.55±0.01 | 0.43±0.03 |
| | 2,2,4,6,6-pentamethylheptane | 0.56±0.01 | - | 0.35±0.02 | - | 0.98±0.01 | 1.96±0.06 | 0.91±0.03 | 2.31±0.05 | - | 2.37±0.26 | 1.23±0.03 |
| | 2,3,5,8-Tetramethyldecane | - | - | - | - | - | - | - | - | 0.59±0.07 | 0.39±0.02 | - |
| | 2,3-dimethyldodecane | - | - | - | 0.17±0.06 | - | - | - | 0.34±0.01 | - | 0.36±0.02 | - |
| | 2,4-dimethylundecane | - | - | - | - | - | - | - | - | 0.38±0.04 | 0.58±0.01 | - |
| Hydrocarbon | 2,5-Dimethylundecane | - | 0.34±0.09 | 0.01±0.04 | 0.37±0.02 | - | 0.55±0.21 | 1.36±0.01 | 0.84±0.02 | 0.80±0.02 | 1.11±0.05 | 0.80±0.04 |
| | 2,6,10,15-Tetramethylheptadecane | 0.76±0.33 | 1.99±0.43 | 1.50±0.01 | 1.54±0.05 | 0.69±0.02 | - | 1.92±0.10 | 0.94±0.28 | 1.39±0.45 | 1.48±0.10 | 0.55±0.04 |
| | 2,6,10-trimethyl pentadecane | 0.64±0.44 | 3.74±0.06 | 0.73±0.04 | 4.06±0.07 | 0.57±0.24 | 3.31±0.14 | 0.30±0.14 | 3.20±0.06 | 0.96±0.29 | 4.09±0.06 | 1.65±0.37 |
| | 2-cyclohexyl eicosane | - | - | - | - | 0.29±0.01 | 0.34±0.03 | - | 0.34±0.01 | 0.59±0.02 | 0.70±0.01 | - |
| | 2-cyclohexyldodecane | 0.20±0.03 | 0.40±0.01 | - | 0.40±0.01 | - | 0.22±0.07 | 0.57±0.06 | - | 0.46±0.02 | 0.42±0.03 | - |
| | 2-methyl-6-propyl-dodecane | - | - | 0.30±0.02 | 0.41±0.02 | 0.45±0.01 | 0.84±0.07 | 0.71±0.15 | 0.48±0.02 | 0.88±0.02 | 1.01±0.05 | 0.88±0.03 |
| | 2-Methyloctadecane | 0.04±0.01 | - | - | 0.27±0.07 | 0.52±0.04 | - | 0.41±0.02 | 0.32±0.02 | - | - | - |
| | 2-Methyltetracosane | 0.04±0.01 | - | 0.38±0.01 | 0.21±0.03 | 0.51±0.03 | 0.38±0.01 | 2.61±0.17 | - | 0.37±0.06 | 0.46±0.05 | 0.54±0.17 |

**Table S1.** (continue).

| Compound Class | Compound Name | Relative content of compounds/% | | | | | | | | | | |
|---|---|---|---|---|---|---|---|---|---|---|---|---|
| | | raw wheat | 3d-EG | 3d-CK | 9d-EG | 9d-CK | 17d-EG | 17d-CK | 22d-EG | 22d-CK | 40d-EG | 40d-CK |
| | 2-Methyldodecane | - | 0.30±0.06 | 0.39±0.03 | 0.62±0.13 | 0.50±0.01 | - | 0.94±0.03 | - | - | 0.86±0.09 | - |
| | 2-Methylhexadecane | 0.28±0.19 | 0.81±0.04 | 0.57±0.01 | 0.63±0.09 | 0.91±0.03 | 0.94±0.03 | 0.79±0.19 | 0.78±0.01 | 0.82±0.15 | 1.01±0.04 | 0.74±0.17 |
| | 2-Methylheptadecane | 0.09±0.02 | 0.22±0.01 | 0.15±0.02 | 0.23±0.01 | 0.37±0.05 | - | - | 0.27±0.02 | 0.50±0.01 | 0.39±0.01 | 0.29±0.01 |
| | 2-Methyl Thirdecane | 0.28±0.11 | 0.71±0.03 | 0.30±0.03 | 0.49±0.01 | 0.36±0.03 | 0.56±0.03 | 0.55±0.18 | 0.55±0.01 | 0.41±0.01 | 0.76±0.01 | 0.47±0.02 |
| | 2-Methylpentadecane | 0.10±0.05 | 0.84±0.05 | - | - | 0.02±0.01 | - | - | 0.68±0.09 | - | 0.82±0.07 | - |
| | 3,5-Dimethyldodecane | 0.21±0.02 | - | - | - | - | - | 0.43±0.01 | - | - | 0.80±0.01 | - |
| | 3,6-Dimethyldecane | 0.22±0.19 | 0.92±0.05 | - | 0.62±0.04 | 0.02±0.01 | 0.81±0.04 | - | 0.97±0.02 | - | 1.00±0.12 | - |
| | 3-Methyldodecane | - | - | - | - | - | 0.77±0.09 | 0.50±0.07 | - | 0.72±0.02 | 0.98±0.01 | 0.63±0.05 |
| | 3-Methylheptadecane | - | - | 1.02±0.01 | 0.32±0.01 | 0.44±0.01 | 0.33±0.01 | 0.42±0.08 | 0.34±0.01 | 0.46±0.04 | 0.42±0.01 | 0.45±0.07 |
| | 3-Methyl Thirdecane | 0.29±0.24 | 0.68±0.05 | 1.21±0.01 | 0.48±0.01 | 0.48±0.10 | 0.58±0.04 | - | 0.57±0.01 | 0.40±0.07 | 0.78±0.01 | 0.80±0.09 |
| | 3-Methyltetradecane | 0.46±0.31 | 2.36±0.03 | 0.71±0.01 | 2.17±0.03 | 1.22±0.10 | 1.98±0.04 | 1.06±0.03 | 1.69±0.07 | 1.31±0.18 | 2.16±0.00 | 0.86±0.23 |
| | 3-Methylpentadecane | 0.24±0.15 | 0.94±0.00 | 0.59±0.01 | 0.97±0.01 | 0.60±0.02 | 0.77±0.02 | 0.48±0.03 | 0.71±0.01 | 0.72±0.21 | 0.85±0.01 | 0.53±0.07 |
| | 3-Methylundecane | - | - | 0.39±0.01 | - | 0.55±0.02 | 0.49±0.05 | 0.61±0.06 | 0.60±0.04 | 0.65±0.06 | 0.54±0.02 | - |
| | 4,4-Dimethylundecane | - | - | - | - | - | - | - | 0.39±0.03 | - | 0.41±0.05 | - |
| | 4,6-Dimethyldodecane | 0.19±0.16 | 1.10±0.06 | 0.54±0.02 | 0.63±0.01 | 0.38±0.02 | 0.69±0.04 | 1.75±0.70 | 1.08±0.03 | 1.17±0.28 | 1.75±0.16 | 0.62±0.13 |
| | 4-cyclohexyl-13decane | - | - | 0.24±0.03 | 0.35±0.02 | 0.60±0.02 | 0.26±0.06 | - | 0.35±0.05 | 0.31±0.06 | 0.50±0.01 | 0.22±0.09 |
| | 4-Methyldecane | - | - | 0.26±0.01 | - | 0.27±0.01 | - | 0.29±0.03 | 0.25±0.06 | 0.17±0.01 | 0.19±0.09 | 0.18±0.06 |

**Table S1.** (continue).

| Compound Class | Compound Name | Relative content of compounds/% | | | | | | | | | | |
|---|---|---|---|---|---|---|---|---|---|---|---|---|
| | | raw wheat | 3d-EG | 3d-CK | 9d-EG | 9d-CK | 17d-EG | 17d-CK | 22d-EG | 22d-CK | 40d-EG | 40d-CK |
| | 4-Methyldodecane | - | - | - | - | - | 0.36±0.03 | 0.40±0.05 | - | 0.38±0.06 | 0.71±0.03 | 0.60±0.19 |
| | 4-Methyl Thirdecane | - | - | - | - | - | - | - | - | - | 0.34±0.02 | - |
| | 4-Methyltetradecane | - | - | - | 0.42±0.03 | - | 0.22±0.04 | - | 0.28±0.03 | - | 0.50±0.04 | 0.18±0.07 |
| | 4-Methylpentadecane | - | - | 0.15±0.01 | 0.48±0.01 | 0.27±0.08 | 0.41±0.01 | - | 0.36±0.01 | - | 0.38±0.01 | - |
| | 5-propyldecane | - | 0.75±0.35 | - | 0.71±0.08 | - | 0.55±0.05 | - | 0.55±0.10 | - | 1.00±0.16 | - |
| | 5-Methyl Thirdecane | - | - | 0.25±0.01 | 0.51±0.04 | 0.28±0.09 | 0.55±0.04 | - | 0.44±0.04 | - | 0.60±0.07 | - |
| | 5-Methyltetradecane | 0.10±0.07 | 0.63±0.06 | 0.79±0.01 | 0.47±0.07 | 0.26±0.01 | 0.52±0.16 | - | 0.29±0.01 | 0.29±0.05 | 0.48±0.01 | 0.47±0.01 |
| | 5-Methylundecane | - | - | 0.02±0.01 | - | 0.32±0.13 | 0.23±0.07 | 0.18±0.04 | 0.20±0.03 | - | 0.29±0.03 | 0.31±0.01 |
| | 5-ethyl-2-methylheptane | - | - | - | - | - | - | - | 0.29±0.02 | - | 0.84±0.01 | - |
| | 6-Methyldodecane | - | - | - | - | - | 0.54±0.24 | - | 0.54±0.04 | 0.75±0.06 | 1.11±0.01 | 0.78±0.02 |
| | 6-Methyl Thirdecane | - | - | 0.49±0.13 | 1.20±0.34 | 1.73±0.37 | 3.22±0.25 | 2.78±0.57 | 1.56±0.43 | 2.91±0.23 | 2.28±0.48 | 2.30±0.23 |
| | 7-Methylhexadecane | - | - | - | 0.49±0.05 | - | - | - | 0.58±0.09 | - | - | - |
| | 7-Methylheptadecane | - | - | - | 0.75±0.02 | 0.84±0.03 | 0.83±0.09 | 0.55±0.09 | 0.65±0.05 | 1.01±0.07 | 0.94±0.02 | 1.14±0.19 |
| | 8-Hexopentadecane | 0.23±0.01 | 0.68±0.06 | 0.70±0.01 | 0.73±0.05 | 0.77±0.03 | 0.85±0.03 | 0.55±0.04 | 0.77±0.03 | - | 0.78±0.05 | 0.91±0.20 |
| | 8-Methylheptadecane | 0.28±0.06 | 0.96±0.01 | 1.10±0.01 | 1.04±0.02 | 1.06±0.15 | 0.82±0.05 | - | 0.84±0.07 | 0.70±0.08 | 1.20±0.08 | - |
| | 9-Methyl-19decane | - | - | - | 0.81±0.02 | - | - | - | - | - | 0.65±0.03 | - |
| | d-limonene | 0.28±0.19 | 0.29±0.01 | 0.45±0.01 | 0.34±0.00 | 1.06±0.13 | 1.43±0.19 | 1.38±0.19 | 0.74±0.06 | 0.71±0.05 | - | 0.58±0.02 |
| | $\alpha$- Pinene | 0.18±0.07 | 1.02±0.18 | 1.58±0.05 | 1.74±0.17 | 1.58±0.20 | 2.12±0.36 | 2.29±1.31 | 2.62±0.11 | 0.94±0.03 | 1.06±0.04 | 0.90±0.01 |
| | Octacosane | - | 0.62±0.07 | 0.26±0.03 | - | - | - | 1.29±0.14 | - | 0.55±0.10 | 0.65±0.13 | 1.74±0.12 |
| | Hexacosane | - | - | 0.83±0.01 | 0.70±0.07 | 0.79±0.12 | 0.62±0.08 | 0.84 | 0.91±0.09 | - | 0.77±0.07 | 0.99±0.17 |

**Table S1.** (continue).

| Compound Class | Compound Name | Relative content of compounds/% | | | | | | | | | | |
|---|---|---|---|---|---|---|---|---|---|---|---|---|
| | | raw wheat | 3d-EG | 3d-CK | 9d-EG | 9d-CK | 17d-EG | 17d-CK | 22d-EG | 22d-CK | 40d-EG | 40d-CK |
| | Tetracosane | 0.19±0.05 | 0.95±0.39 | 1.20±0.01 | 0.67±0.22 | 1.53±0.17 | 1.10±0.07 | 1.81±0.61 | 1.12±0.04 | 1.64±0.65 | 1.23±0.13 | 0.89±0.51 |
| | Eicosane | - | - | 1.29±0.01 | 0.88±0.08 | 0.84±0.11 | 0.50±0.07 | - | 0.68±0.07 | 0.80±0.09 | 1.19±0.09 | 1.58±0.30 |
| | Pentacosane | 0.68±3.19 | 1.43±0.31 | - | 1.23±0.18 | - | 0.71±0.09 | - | 1.22±0.27 | 0.88±0.07 | 0.81±0.06 | 1.00±0.10 |
| | Heneicosane | 1.24±0.27 | 0.65±0.01 | 0.53±0.01 | 0.91±0.05 | 1.13±0.04 | 0.90±0.04 | 0.98±0.08 | 0.88±0.10 | 1.88±0.85 | - | 0.62±0.19 |
| | Decane cyclohexane | 0.09±0.01 | - | - | 0.40±0.01 | - | 0.25±0.12 | 0.68±0.05 | 0.33±0.01 | 0.27±0.03 | - | - |
| | Decane | 0.13±0.02 | 0.50±0.08 | 0.97±0.01 | 0.64±0.02 | 1.61±0.05 | - | 1.71±0.39 | 1.45±0.23 | 1.16±0.27 | 0.50±0.01 | 1.19±0.09 |
| | Cyclohexane (1,3-dimethylbutyl) | - | - | - | 0.39±0.02 | - | 0.36±0.02 | 0.31 | 0.28±0.01 | - | 0.39±0.01 | - |
| | Cyclohexane (1-octylnonyl) | - | 0.21±0.03 | 0.14±0.01 | 0.22±0.03 | - | - | 0.22±0.06 | - | - | 0.32±0.01 | 0.38±0.05 |
| | Cyclohexane, (1-hexyltetradecyl) | - | 0.25±0.03 | - | 0.25±0.09 | 0.34±0.07 | - | - | 0.17±0.04 | 0.34±0.01 | 0.33±0.10 | - |
| | Thirty six alkanes | 0.65±0.01 | - | - | - | - | 4.50±0.29 | - | 0.48±0.04 | - | - | - |
| | Triacontane | 0.05±0.01 | - | - | - | - | 0.77±0.03 | - | - | 0.55±0.07 | - | 0.58±0.13 |
| | Squalene | 0.67±0.36 | - | 0.64±0.01 | 0.23±0.04 | 1.04±0.20 | 5.97±0.10 | 0.97±0.30 | 3.03±0.36 | 0.35±0.02 | 4.66±0.01 | - |
| | Octadecane | 0.65±0.03 | 2.07±0.07 | 2.02±0.03 | 2.16±0.07 | 2.75±0.04 | 2.55±0.19 | 3.15±0.45 | 2.06±0.11 | 2.26±0.39 | 2.46±0.11 | 2.16±0.24 |
| | Dodecane | 0.72±0.50 | 1.49±0.11 | 1.03±0.03 | 1.58±0.10 | 0.55±0.03 | 1.75±0.08 | 1.05±0.53 | 3.13±0.07 | 1.72±0.02 | 5.29±0.03 | 1.39±0.32 |
| | Dodecane, 2,6,10-trimethyl pentadecane | 0.31±0.07 | 0.61±0.02 | - | 0.63±0.12 | - | - | - | 0.54±0.01 | - | 0.91±0.02 | 1.95±0.23 |
| | Dodecane, 2,6,11-trimethyl | - | - | - | 0.40±0.01 | - | 0.44±0.02 | - | - | - | - | 0.60±0.09 |

**Table S1** (continue)

| Compound Class | Compound Name | Relative content of compounds/% | | | | | | | | | | |
|---|---|---|---|---|---|---|---|---|---|---|---|---|
| | | raw wheat | 3d-EG | 3d-CK | 9d-EG | 9d-CK | 17d-EG | 17d-CK | 22d-EG | 22d-CK | 40d-EG | 40d-CK |
| | Dodecane, 2,7,10-trimethyl | 0.16±0.07 | 0.59±0.02 | - | 0.38±0.03 | - | 0.68±0.05 | - | 0.53±0.01 | - | 0.94±0.05 | - |
| | Decadecane | 0.10±0.04 | - | 0.83±0.11 | 0.78±0.05 | 0.61±0.10 | - | 1.38±0.13 | 0.73±0.10 | - | 1.20±0.02 | 1.10±0.09 |
| | Hexadecane | 1.79±1.33 | 11.37±0.05 | 1.42±0.08 | 11.32±0.10 | 2.33±0.36 | 8.50±0.46 | 3.48±0.96 | 8.22±0.20 | 3.48±0.47 | 11.18±0.08 | 3.17±0.29 |
| | Cetane, 2,6,10,14-tetramethyl | 0.18±0.01 | 2.48±0.23 | 1.27±0.01 | 1.61±0.13 | 1.09±0.02 | 1.26±0.09 | 1.26±0.15 | 1.35±0.07 | 1.05±0.21 | 1.73±0.04 | 1.35±0.35 |
| | Heptadecane | 1.27±0.92 | 9.10±0.06 | 1.91±0.10 | 9.83±0.10 | 2.74±0.49 | 7.77±0.47 | 3.01±0.20 | 7.79±0.21 | 3.23±0.07 | 11.48±0.13 | 5.76±0.80 |
| | Tridecane | 0.66±0.52 | 1.95±0.04 | 1.29±0.03 | 1.62±0.02 | 2.69±0.05 | 1.63±0.06 | 1.10±0.73 | 1.92±0.05 | 1.94±0.29 | 7.82±0.03 | 1.72±0.78 |
| | Tetradecane | 0.37±0.29 | 3.04±2.15 | - | 1.22±0.39 | 2.74±0.21 | 3.80±0.27 | - | 2.84±0.38 | 0.55±0.10 | 4.48±0.52 | - |
| | Pentadecane | 2.26±1.66 | 11.56±0.40 | 1.71±0.07 | 10.12±0.28 | 1.78±0.26 | 7.50±0.25 | 1.51±0.67 | 6.66±0.25 | 1.26±0.45 | 10.13±0.20 | 1.21±0.46 |
| | Pentadecane, 2,6,10,14-tetramethyl | 0.50±0.32 | 2.82±0.01 | 2.64±0.05 | 3.14±0.05 | 2.26±0.39 | 2.88±0.18 | 2.78±0.01 | 2.46±0.08 | 2.87±0.61 | 3.74±0.45 | 2.08±0.60 |
| | Undecane | 0.35±0.22 | 0.70±0.04 | 0.42±0.03 | 0.91±0.05 | 2.78±0.12 | 1.40±0.08 | 3.22±0.44 | 2.82±0.09 | 2.72±0.42 | 0.92±0.05 | 2.37±0.06 |
| | Tetratetracontane | 0.97±0.38 | - | - | 0.11±0.01 | - | - | - | 0.28±0.04 | - | 0.22±0.01 | - |
| Alcohols | 2-ethylhexanol | 1.33±0.93 | 3.96±0.12 | 1.03±0.11 | 2.90±0.17 | 1.79±0.38 | 3.59±0.06 | 1.52±0.07 | 2.94±0.09 | 1.25±0.03 | 3.37±0.04 | 1.09±0.07 |
| | 3,5-Dimethylhexanol | 0.36±0.13 | 1.21±0.03 | 0.70±0.01 | - | 0.49±0.05 | 1.16±0.07 | 0.41±0.02 | 0.61±0.01 | 0.34±0.02 | 0.81±0.07 | 0.35±0.03 |
| | Phytosterol | 0.27±0.10 | 0.30±0.18 | - | 0.25±0.01 | - | 0.31±0.01 | - | 0.36±0.02 | - | - | - |
| | 3,7,11,15-Tetramethyl-1-hexadecanol | - | - | - | - | - | 0.45±0.15 | - | - | - | 0.85±0.03 | - |
| | 2-Butyl-1-octanol | - | 0.42±0.03 | - | - | - | - | - | - | - | 0.54±0.02 | - |

**Table S1** (continue)

| Compound Class | Compound Name | Relative content of compounds/% | | | | | | | | | | |
|---|---|---|---|---|---|---|---|---|---|---|---|---|
| | | raw wheat | 3d-EG | 3d-CK | 9d-EG | 9d-CK | 17d-EG | 17d-CK | 22d-EG | 22d-CK | 40d-EG | 40d-CK |
| | 3,4,4-Trimethyl-3-pentanol | - | 0.47±0.04 | 0.01±0.04 | - | - | 0.64±0.06 | - | 0.59±0.02 | 0.61±0.02 | 0.63±0.03 | - |
| | 3-methyl-3-heptanol | - | 0.48±0.08 | - | - | 0.62±0.03 | 0.66±0.05 | - | 0.59±0.04 | 0.57±0.01 | 0.66±0.02 | 0.59±0.07 |
| Aldehydes | Benzaldehyde | 0.39±0.10 | 1.55±0.05 | 0.20±0.04 | 1.50±0.02 | 0.81±0.10 | 1.45±0.03 | 0.72±0.08 | 2.47±0.07 | 0.74±0.03 | 2.10±0.02 | 0.57±0.05 |
| | Decanal | 0.23±0.15 | 1.15±0.02 | 1.41±0.01 | 1.23±0.02 | 0.52±0.03 | 0.50±0.02 | 2.04±0.60 | 1.04±0.03 | 2.47±0.19 | 0.41±0.01 | 3.96±0.63 |
| | Nonanal | 0.45±0.35 | 1.81±0.06 | 2.87±0.01 | 1.65±0.01 | 1.21±0.06 | 0.92±0.03 | 2.20±0.43 | 1.62±0.08 | 2.69±0.01 | 0.90±0.05 | 3.83±0.48 |
| | Undecylaldehyde | - | 0.19±0.05 | - | 0.19±0.01 | - | - | - | - | - | - | 0.32±0.02 |
| | Dodecanal | - | 0.58±0.04 | 0.72±0.01 | 0.26±0.07 | - | - | 0.36±0.02 | - | - | 0.44±0.11 | 0.54±0.01 |
| Ketones | 5-Heptene-2-one, 6-methyl | 0.40±0.22 | 0.30±0.08 | 0.84±0.01 | 0.47±0.07 | 0.20±0.07 | - | 0.92±0.03 | 0.55±0.03 | 0.63±0.02 | 0.82±0.04 | 0.70±0.01 |
| | 3-ethyl-3-methyl-2-pentanone | - | 0.64±0.01 | - | 0.78±0.06 | - | 0.62±0.01 | - | 1.15±0.78 | - | - | - |
| | Acetophenone | - | 0.77±0.02 | - | - | - | - | - | - | 0.74±0.08 | 0.80±0.11 | 0.70±0.04 |
| | 1-methyl-3,6-diaza-9-one | - | 0.51±0.14 | 0.18±0.01 | 0.74±0.02 | - | 0.70±0.06 | - | 0.74±0.10 | 0.12±0.04 | 0.87±0.02 | 0.05±0.01 |
| | 5,5-Dimethyl-3-vinylcyclohex-2-en-1-one | - | - | - | 1.61±0.19 | - | 1.24±0.23 | - | - | - | - | - |
| | 2-hexanone, 3,3-dimethyl | - | - | - | - | - | - | - | 0.77±0.02 | - | 0.53±0.04 | - |
| | 1- (4-ethylphenyl) ethanone | - | - | - | - | - | 0.87±0.12 | - | - | - | - | - |
| | 1- (3-Butylepoxyethanyl) ethanone | - | - | - | - | - | - | - | 0.75±0.03 | - | - | - |

**Table S1** (continue)

| Compound Class | Compound Name | Relative content of compounds/% | | | | | | | | | | |
|---|---|---|---|---|---|---|---|---|---|---|---|---|
| | | raw wheat | 3d-EG | 3d-CK | 9d-EG | 9d-CK | 17d-EG | 17d-CK | 22d-EG | 22d-CK | 40d-EG | 40d-CK |
| | 3-Octanone | - | - | - | - | - | - | - | - | - | 0.93±0.01 | - |
| Esters | Ethyl hexanoate | 0.57±0.46 | 0.98±0.11 | 1.03±0.01 | 1.10±0.01 | 1.18±0.03 | 1.66±0.11 | 1.26±0.13 | 1.21±0.03 | - | 1.46±0.02 | - |
| | Dibutyl phthalate | 3.13±0.69 | 7.33±0.36 | 3.75±0.09 | 5.69±0.28 | 3.47±0.40 | 6.95±0.38 | 4.29±1.37 | 7.22±0.39 | 4.58±0.67 | 7.62±0.57 | 4.39±0.71 |
| | Dimethyl phthalate | 0.68±0.24 | 1.24±0.08 | 0.46±0.01 | 1.45±0.03 | - | 1.43±0.04 | 1.16±0.14 | 1.00±0.02 | 0.62±0.02 | 0.81±0.04 | - |
| | Bis (2-ethylhexyl) phthalate | 0.43±0.25 | - | - | - | - | 0.48±0.11 | - | 0.45±0.01 | - | - | - |
| | 2-Methylpropionic acid 3-hydroxy-2,4,4-trimethylpentyl ester | - | 0.26±0.02 | - | 0.23±0.08 | - | - | - | - | - | - | - |
| | Amino acid, (1-phenylethyl) -3-methyloctyl ester | - | 0.64±0.01 | - | 0.18±0.01 | - | - | - | 0.56±0.01 | - | 0.60±0.09 | - |
| | Diisooctyl phthalate | - | 0.39±0.07 | 2.29±0.01 | 0.29±0.08 | 0.49±0.05 | - | 0.38±0.04 | 0.66±0.08 | 0.58±0.02 | 0.41 | 0.49±0.04 |
| | Amino acid（ α-Methylbenzyl) -1-ethyl-1-methylhexyl ester | - | 0.19±0.01 | - | 0.50±0.12 | - | 0.67±0.07 | - | 0.53±0.14 | - | 0.66±0.48 | - |
| | Di-n-octyl phthalate | 0.45±0.03 | - | 0.74±0.08 | - | 1.01±1.57 | 0.36 | 0.42±0.03 | - | 0.52±0.04 | 0.2±0.02 | - |
| | (-) -1-methylcholanthrene | 0.31±0.04 | - | - | 1.98±0.02 | - | 1.67±0.05 | - | 1.04±0.13 | - | 1.39±0.05 | - |
| | 1,2,3-trimethylbenzene | 1.03±0.02 | 1.10±0.01 | 0.79±0.03 | 1.52±0.11 | 0.16±0.03 | 1.21±0.08 | 0.16±0.69 | 2.83±0.33 | 0.90±0.12 | 1.75±0.06 | 0.70±0.16 |
| | 1,4-Bis [2- (3-chlorophenoxy) ethyl] piperazine | 0.30±0.02 | - | 0.41±0.01 | 1.82±0.28 | 0.78±0.01 | - | 0.81±0.06 | 1.20±0.01 | 0.77±0.04 | 1.50±0.12 | 1.44±0.12 |

**Table S1** (continue)

| Compound Class | Compound Name | Relative content of compounds/% | | | | | | | | | | |
|---|---|---|---|---|---|---|---|---|---|---|---|---|
| | | raw wheat | 3d-EG | 3d-CK | 9d-EG | 9d-CK | 17d-EG | 17d-CK | 22d-EG | 22d-CK | 40d-EG | 40d-CK |
| Others | 1-Iodidecane | 0.35±0.27 | - | - | 1.28±0.10 | 0.61±0.12 | - | 0.83±0.10 | 0.72±0.14 | 0.50±0.10 | 1.03±0.03 | 0.67±0.09 |
| | 1-Chlorotetradecane | 0.14±0.02 | - | 0.06±0.01 | 0.86±0.01 | 0.92±0.14 | 0.73±0.09 | 0.43±0.01 | 0.73±0.09 | - | 1.04±0.07 | 0.78±0.29 |
| | 1-ethyl-3-methylbenzene | - | 0.79±0.30 | 1.01±0.02 | 1.03±0.07 | 3.00±0.52 | - | 1.33±0.37 | 1.87±0.29 | 1.99±0.47 | 0.69±0.07 | 1.30±0.01 |
| | 2-Nitrohexane | - | - | 1.04 | 1.04±0.05 | - | 0.82±0.11 | 1.51±0.12 | 2.14±0.29 | 0.30±0.01 | 1.41±0.29 | 0.30±0.01 |
| | 2-bromotetradecane | - | - | - | - | - | - | - | - | 0.12±0.02 | 0.61±0.03 | - |
| | Benzene (1-methylethyl) | - | 1.67±0.13 | 0.38±0.06 | 1.60±0.10 | - | 1.09±0.12 | 0.57±0.03 | 1.39±0.31 | - | - | - |
| | Benzothiazole | - | 0.61±0.09 | - | 0.52±0.10 | - | 0.57±0.09 | - | 0.60±0.09 | - | - | - |
| | phenol | - | 3.62±1.09 | - | 3.19±0.16 | - | 3.88±0.95 | - | 3.29±0.10 | 0.96±0.01 | 3.95±1.90 | 0.64±0.27 |
| | Paraoxane | 0.54±0.09 | - | 0.82±0.01 | 0.44±0.01 | 0.82±0.13 | - | - | 0.76±0.08 | - | - | - |
| | Trimethylbenzene | - | 0.46±0.14 | - | 0.44 ±0.01 | 0.78±0.17 | 1.14±0.08 | 0.31±0.06 | 1.31±0.22 | - | 0.61±0.05 | - |
| | naphthalene | 0.52±0.04 | 1.14±0.05 | 0.29±0.02 | 1.26±0.03 | 0.79±0.15 | 1.17±0.01 | 0.09±0.01 | 1.48±0.04 | 0.37±0.04 | 1.03±0.05 | 0.89±0.21 |
| | Triphenylphosphine oxide | 0.52±0.01 | - | 0.19±0.05 | 0.54±0.04 | 2.02±0.11 | - | 6.94±0.19 | 6.24±3.05 | - | 1.88±0.12 | 3.84±0.21 |
| | Tetramethylbenzene | 0.75±0.01 | - | - | 0.72±0.03 | 0.62±0.03 | 0.45±0.02 | 0.74±0.03 | 0.89±0.33 | 0.65±0.10 | - | - |
| | 1,2,3,5-tetramethylbenzene | - | 0.72±0.10 | 0.45±0.01 | 0.56±0.01 | - | 0.63±0.01 | 1.17±0.54 | 0.79±0.02 | 0.78±0.23 | 1.19±0.16 | 0.36±0.04 |
| | 1-Methylethylbenzene | - | - | - | 0.45±0.03 | - | 0.21±0.04 | - | 0.68±0.04 | - | 0.69±0.07 | - |

†. CK-3d,9d,17d,22d,40d refer to wheat samples stored naturally for 3,9,17,22,40 days and EG-3d,9d,17d,22d,40d refers to wheat samples after 3,9,17,22,40 days of infestation by rice weevil.
